# A case-control study on *Chlamydia psittaci* pneumonia and legionella pneumonia

**DOI:** 10.3389/fmed.2025.1591963

**Published:** 2025-06-24

**Authors:** Ying Gao, Yuan-jie Lin, Wen-long Zhang, Jian-ming Ni, Shu-guang Han, Liang Bao

**Affiliations:** ^1^Department of Respiratory and Critical Care Medicine, The Wuxi Second People’s Hospital, Wuxi, China; ^2^Department of Radiology, The Wuxi Second People’s Hospital, Wuxi, China

**Keywords:** *Chlamydia psittaci* pneumonia, *Legionella* pneumonia, LASSO regression, retrospective analysis, clinical characteristics

## Abstract

**Purpose:**

Atypical pathogens (*Chlamydia psittaci* and *Legionella*) are often detected by metagenomic next-generation sequencing (mNGS). However, the two atypical pneumonias are difficult to distinguish. The aim of this study was to retrospectively analyze the two types of atypical pneumonia and use statistics to find points of differentiation for early diagnosis and timely treatment.

**Methods:**

This retrospective study included all confirmed cases of two types of atypical pneumonia in our institution. The data collected and analyzed included epidemiological, clinical, laboratory, and radiological features.

**Results:**

The study included 84 patients, 63 with *Chlamydia psittaci (C. psittaci)* pneumonia, 21 with *Legionella* pneumonia. (1) Up to 61.9% of patients with *C. psittaci* pneumonia and *Legionella* pneumonia had high fevers. More than 90% of patients with *Legionella* pneumonia had a cough score ≥ 3. *Legionella* pneumonia patients experienced more severe coughing, chest tightness and shortness of breath symptoms than *C. psittaci* pneumonia patients (both, *p* < 0.01). (2) Consolidation, bronchial insufflation, ground-glass opacities, and pleural effusion are the most common chest CT signs of *C. psittaci* pneumonia and *Legionella* pneumonia. *Legionella* pneumonia was more likely to cause ground-glass opacities in the upper left lobe than *C. psittaci* pneumonia (*p* = 0.05). There was no statistical difference in other CT findings. (3) *C. psittaci* pneumonia and *Legionella* pneumonia were identified by leukocytes, lymphocytes ratio, NLR, blood glucose, cough, chest tightness and shortness of breath. They had AUC’ s of 0.810, 0.709, 0.728, 0.724, 0.795, 0.675, and respective 95% CI’ s of 0.716–0.907, 0.60 5–0.832, 0. 566–0.838, 0.604–0.831, 0.696–0.869, 0.574–0.784; all statistically significant (all *P* < 0.05; < 0.001, 0.003, 0.008, 0.006, < 0.001, 0.017, respectively). (4) 69.8%, 80.9% of each patients took two or more antibiotics simultaneously before diagnosis, but the difference was not statistically significant (*p* = 0.32). Some patients received more than four antibiotics, most commonly *Legionella* pneumonia (23.8%) (*p* = 0.01).

**Conclusion:**

Clinicians should consider atypical pneumonia, particularly *C. psittaci* and *Legionella* pneumonia, when patients present with high fever and chest CT scans showing consolidation accompanied by bronchial insufflation, ground-glass opacities, and pleural effusion. Initially, clinicians can differentiate between the two types of pneumonia based on symptoms (e.g., cough severity, chest tightness and shortness of breath), imaging features (e.g., GGO in the left upper lobe), and laboratory markers (e.g., glucose, leukocytes, NLR, and lymphopenia). This allows for the optimization of antibiotic choices and the reduction of unnecessary multidrug combinations, which can improve prognosis and reduce the risk of drug resistance.

## Background

A leading cause of infectious disease burden worldwide is community-acquired pneumonia (CAP) (1). The causes of CAP are now better understood thanks to improvements in the sensitivity, availability and affordability of molecular pathogen testing over the past decade (1). In a multi-center prospective study involving 17 hospitals and approximately 275 patients in China, *Legionella pneumophila* accounted for 11.3%, and *Chlamydia Psittaci (C. psittaci)* for 6.8% of severe community-acquired pneumonia (2). Both pathogens are capable of causing atypical CAP, which accounts for about 15% of all cases. The importance of atypical pneumonias is not their frequency but their difficult diagnosis and non-responsiveness to β-lactam therapy (3).

Atypical CAP pathogens, *C. psittaci* pneumonia is a zoonotic disease. Its main modes of transmission are contact with secretions (e.g., feces, feathers, and respiratory secretions) or inhalation of aerosols from infected birds. High-risk groups include bird keepers, veterinarians, pet store workers, and poultry processing workers (4, 5). With *Legionella* usually infected Patients through exposure to moist contaminated environments (e.g., hotel showers, air conditioning, etc.). Patients are particularly susceptible to immunosuppression, immunocompromise and the elderly (6). We know that both pathogens cause severe CAP and that their clinical features and imaging signs are similar to typical CAP. Some physicians treat them with multiple antibiotics simultaneously during initial therapy, leading to antibiotic misuse and exacerbating the problem of pathogen resistance and the emergence of superbugs.

Clinically, both start with high fever and cough and can involve multiple systems. Both can have markedly elevated inflammatory indexes, hypokalemia, hyponatremia, hypoproteinemia, markedly elevated lactate dehydrogenase (LDH) and creatine kinase (CK), etc. The specificity of routine laboratory tests is low, and imaging lacks specificity. Most local and municipal hospitals, as well as some tertiary-academic affiliated teaching hospitals, lack pathogen specific detection methods (e.g., psittacosis serologic antibody testing and *Legionella* urinary antigen testing). If patients conceal a history of avian contact or do not have clear environmental exposure, differential diagnosis is difficult and ultimately relies on pathogen testing. However, a literature search revealed that systematic analyses of the clinical features of atypical pneumonia associated with *C. psittaci* and *Legionella* pneumophila infections are rare. In this paper, we retrospectively analyzed 84 cases of atypical pneumonia in patients admitted to our hospital. Using statistical methods, we explored the clinical, laboratory, and imaging characteristics of the two types of pneumonia and searched for their differentiating factors. Our goal was to guide clinicians in diagnosing and differentiating these two diseases and to prevent the misuse of antibiotics.

## Materials and methods

### Enrolled subjects

This retrospective study included 84 patients with atypical pneumonia. Patients were admitted between January 1, 2020, and December 31, 2023, as well as those who were diagnosed and treated at Wuxi Second People’s Hospital. The inclusion diagnostic criteria for atypical pneumonia were as follows: (1) diagnosed with atypical CAP according to the guidelines (7, 8); (2) identification of the *C. psittaci*, and *Legionella* gene fragments through metagenomic next-generation sequencing (mNGS) analysis of the bronchoalveolar lavage fluid (BALF), blood, or Sputum, and met the criteria for a positive mNGS result (9); (3) Positive results from BALF or serum pathogenic IgM antibodies; routine microbiological tests, including blood, sputum, and BALF culture were all negative (10, 11).

This study was conducted according to the principles of the Declaration of Helsinki and approved by the Wuxi Second People’s Hospital (NO.2024, Y-185). All research data were anonymously analyzed.

### Procedures

From patients’ medical records, we collected epidemiological, demographic, clinical, laboratory, management, and outcome data. For missing data or clarification, we contacted attending doctors, other healthcare providers, and patients. All data were checked by two physicians (Ying Gao and Yuan-Jie Lin).

The mNGS testing was primarily undertaken by the Genskey (Shanghai, China), Adicon (Hangzhou, China), Darui Diagnostics (Guangzhou, China), and Shenzhen Huada Gene Technology Co., Ltd. (Shenzhen, China). The following outlines the mNGS assay process and bioinformatics analysis.

Procedure: First, the respiratory samples were broken using a wall breaker and centrifuged. Then, 600 μL of the supernatant was taken for DNA and RNA nucleic acid extraction using a nucleic acid extraction or purification kit (Tianjin Golden Spoon Medical, Jinmu Bei No. 20210111). Next, RNA was reverse transcribed to detect both DNA and RNA pathogens. Then, target enrichment of pathogens and library preparation were performed using the Universal Targeted Enrichment Kit for Pathogenic Microorganisms (Universal Kit for Sequencing Reaction Preparation, Tianjin Gold Key Medical, Jinmu Bei No. 20210608). The constructed libraries were pooled and sequenced using the MGISEQ-2000 platform with a 50 bp single-end sequencing strategy.

Bioinformatics analysis: Sequencing raw data were converted into fq format capable of data processing using bcl2fastq software. Data were quality controlled using fastp (v0.23.2) to remove low-quality, low-complexity, and short-length sequences, ensuring a Q30 quality score of over 85%. Sequences of host origin were removed after quality control using bwa-mem (v0.7.17) software for comparison with the human reference genome (T2T-CHM13v2.0). The remaining sequences were compared to the pathogen amplicon reference database using bwa-mem (v0.7.17) for pathogen identification. Finally, pathogens were threshold filtered to obtain final pathogen results.

On admission, samples of blood, sputum, or endotracheal aspirates were collected to ascertain the possible causative bacteria or fungi. All patients received chest computed tomography (CT) scans and laboratory testing.

Influenza-Like Symptoms (ILS) are defined as having at least one of the following signs or symptoms recorded in the medical record as the chief complaint: chills, fever, upper respiratory infection, cough, sore throat, runny nose, congestion, headache, or fatigue (12).

The degree of cough was evaluated by a semi-quantitative cough strength score (SCSS) (13, 14). It is scored as 0 = no cough; 1 = no cough, but the airflow in the mouth is audible; 2 = weak (barely) audible cough; 3 = clearly audible cough; 4 = strong cough; 5 = continuous strong cough.

The consolidation and ground-glass opacity scores on chest CT were evaluated using CT visual quantitative evaluation, which was based on summing up the acute lung inflammatory lesions involving each lobe, and scored as 0 (0%), 1 (1–25%), 2 (26–50%), 3 (51–75%), or 4 (76–100%), respectively. Summing the five lobe scores results in the total score (15).

### Outcomes

We described the clinical characteristics, demographics, primary medical conditions, clinical signs and symptoms, chest CT signs, laboratory results, bacteriology, comorbidities, treatment, and outcome of both types of atypical pneumonia. Further, we found meaningful differences between *C. psittaci* pneumonia and *Legionella* pneumonia based on statistical analysis.

### Statistical analysis

Continuous measures were depicted as mean ± standard deviation (SD) if they were normally distributed, otherwise, depicted as median (interquartile range, IQR). Categorical variables were depicted as counts (%). Statistical analysis was performed separately using Kruskall-Wallis, Pearson, and Wilcoxon. For the statistical analyses of *C. psittaci* pneumonia and *Legionella* pneumonia, variables were screened by LASSO regression analysis combined with Random Forrest Classifier for variable significance analysis. Then the variables were analyzed by the receiver operating characteristic curve. R software 4.2.3 were applied for all analyses.

## Results

### Patient characteristics

84 patients were included: 63 with *C. psittaci pneumonia*, and 21 with *Legionella* pneumonia. There was no significant difference in age and sex between the two types of pneumonia. Detailed clinical information is presented in Table 1 for all patients.

**TABLE 1:** Demographics, baseline characteristics, and clinical outcomes of 84 patients.

	*C. psittaci* pneumonia	Legionella	*p*-value
	***N* = 63^1^**	***N* = 21^1^**	
**Age (years):** median (IQR)	58.0 (46.2—66.0)	62.0 (40.3—70.3)	0.55^3^
**Sex: Female**	28 (44.4%)	5 (23.8%)	0.09^2^
**Exposure history: Yes**	36 (57.1%)	3 (14.2%)	< 0.01^2^
Facial Spa	0 (0.0%)	1 (4.8%)	0.08^2^
Swimming in public baths	0 (0.0%)	1 (4.8%)	0.08^2^
Drowning	0 (0.0%)	1 (4.8%)	0.08^2^
Exposure to pigeon	8 (12.7%)	0 (0.0%)	0.09^2^
Exposure to parrot	23 (36.5%)	0 (0.0%)	< 0.01^2^
Exposure to wild birds	5 (7.9%)	0 (0.0%)	0.18^2^
**Diabetes: Yes**	9 (14.3%)	7 (33.3%)	0.05^2^
**Ketone bodies: Yes**	23 (36.5%)	14 (66.7%)	0.02^2^
**Complications: Yes**	33 (52.4%)	20 (95.2%)	< 0.01^2^
ARDS	2 (3.2%)	1 (4.8%)	0.73^2^
Respiratory failure	10 (15.9%)	11 (52.4%)	< 0.01^2^
Acute liver injury	29 (46.0%)	11 (52.4%)	0.61^2^
Acute renal injury	7 (11.1%)	4 (19.0%)	0.35^2^
Cardiac insufficiency	4 (6.3%)	1 (4.8%)	0.79^2^
Myocardial injury	1 (1.6%)	0 (0.0%)	0.56^2^
Atrial fibrillation	3 (4.8%)	0 (0.0%)	0.31^2^
Pulmonary embolism	1 (1.6%)	0 (0.0%)	0.56^2^
Gastrointestinal bleeding	3 (4.8%)	0 (0.0%)	0.31^2^
Septic shock	1 (1.6%)	0 (0.0%)	0.56^2^
Rhabdomyolysis	1 (1.6%)	0 (0.0%)	0.56^2^
Electrolyte metabolism disorder	45 (71.4%)	20 (95.2%)	0.02^2^
Hypoalbuminemia	38 (60.3%)	12 (57.1%)	0.80^2^
**Antibacterial**			
≥ 2 antibiotics taken simultaneously before diagnosis	44 (69.8%)	17 (80.9%)	0.32^2^
Application of ≥ 4 antibiotics to one patient	3 (4.8%)	5 (23.8%)	0.01^2^
Doxycycline	41 (65%)	2 (9.5%)	< 0.01^2^
Azithromycin	6 (9.5%)	3 (14%)	0.54^2^
Minocycline	1 (1.6%)	0 (0%)	0.56^2^
Moxifloxacin	48 (76%)	14 (67%)	0.39^2^
Nemonoxacin Malate	10 (16%)	5 (24%)	0.41^2^
Levofloxacin	4 (6.3%)	4 (19%)	0.09^2^
Cefotaxime Sodium and Sulbactam Sodium	7 (11%)	1 (4.8%)	0.39^2^
Cefoperazone sulbactam	1 (1.6%)	1 (4.8%)	0.41^2^
Piperacillin tazobactam	21 (33%)	7 (33%)	1.00^2^
Cefpiramide	2 (3.2%)	0 (0%)	0.41^2^
Radix cephalosporin	2 (3.2%)	0 (0%)	0.41^2^
Carbapenems	20 (31.7%)	14 (66.7%)	0.01^2^
Imipenemcilastatin	3 (4.8%)	3 (14%)	0.14^2^
Biapenem	15 (24%)	6 (29%)	0.66^2^
Meropenem	1 ((1.6%)	5 (24%)	< 0.01^2^
Etapenem	1 ((1.6%)	0 (0%)	0.56^2^
Vancomycin	2 (3.2%)	1 (4.8%)	0.73^2^
Ceftazidime Avibactam	0 (0%)	1 (4.8%)	0.08^2^
Tigecycline	1 ((1.6%)	0 (0%)	0.56^2^
Etimicin	0 (0%)	1 (4.8%)	0.08^2^
SMZ	0 (0%)	1 (4.8%)	0.08^2^
FluconTazole	1 (1.6%)	1 (4.8%)	0.41^2^
VoriconTazole	0 (0%)	2 (9.5%)	0.01^2^
Caspofungin	0 (0%)	1 (4.8%)	0.08^2^
**Severe: Yes**	17 (27.0%)	15 (71.4%)	< 0.01^2^
**Mechanical ventilation: Yes**	2 (3.2%)	2 (9.5%)	0.24^2^
**Oxygen therapy: Yes**	28 (44.4%)	15 (71.4%)	0.03^2^
**Vest: Dead**	0 (0.0%)	1 (4.8%)	0.08^2^
**Length of hospitalization (days):** Median ((IQR)	9.0 (7.0—11.0)	13.0 (9.7—15.3)	0.01^3^
**Imaging uptake time (days):** Median ((IQR)	12.0 (8.2—13.1)	13.0 (10.0—13.1)	0.43^3^
**Days of mNGS testing after admission:** Median ((IQR)	2.00 (1.00— 3.00)	2.50 (1.00— 3.00)	0.61^3^

N is the number of non-missing value. ^1^Kruskal-Wallis. ^2^Pearson. ^3^Wilcoxon. ARDS, acute respiratory distress syndrome; IQR, interquartile range.

There was a difference in the exposure history between the two. *C. psittaci* may be associated with contact with parrots/birds, while *Legionella* may cause illness due to contact with contaminated water sources.

The most common complications of *C. psittaci* pneumonia were electrolyte metabolism disorders (71.4%), hypoalbuminemia (60.3%), acute liver injury (46.0%), and respiratory failure (15.9). The incidence of complications in *Legionella* pneumonia was higher (*p* < 0.01), mainly accompanied by electrolyte metabolism disorders (95.2%), hypoalbuminemia (57.1%), respiratory failure (52.4%), acute liver injury (52.4%), and acute kidney injury (19.0%).

We observed that the main clinical manifestations of *C. psittaci* pneumonia were influenza - like symptoms (49.2%), chills (46.0%), high fever (61.9%), mild or no cough (49.2%), and no sputum (60.3%), etc. The main clinical symptoms of *Legionella* pneumonia were chills (33.3%), high fever (61.9%), severe cough (90.5%), expectoration (71.4%), chest tightness and shortness of breath (47.6%), etc.

The most common clinical manifestation of the two was high fever, up to 61.9%. All symptoms are listed in Table 2.

**TABLE 2:** Clinical symptoms of 84 patients.

	*C. psittaci* pneumonia (*N* = 63)	Legionella (*N* = 21)	*p*-value
Peak body temperature (°C): Median (IQR)	39.5 (39.0–40.0)	39.4 (39.0–40.0)	0.91^3^
Fever			0.07^2^
High	39/63 (61.9)	13/21 (61.9)	0.87^2^
Moderate	21/63 (33.3)	6/21 (28.6)	0.69^2^
Low	3/63 (4.8)	0/21 (0.0)	0.30^2^
No	0/63 (0.0)	2/21 (9.5)	0.02^2^
Cough			< 0.01^2^
0	18/63 (28.6)	0/21 (0.0)	0.01^2^
1	1/63 (1.6)	1/21 (4.8)	0.41^2^
2	12/63 (19.0)	1/21 (4.8)	0.12^2^
3	27/63 (42.9)	9/21 (42.9)	1.0^2^
4	4/63 (6.3)	7/21 (33.3)	< 0.01^2^
5	1/63 (1.6)	3/21 (14.3)	0.02^2^
Expectoration: Yes	25/63 (39.7)	15/21 (71.4)	0.01^2^
Sputum character			< 0.01^2^
No	38/63 (60.3)	6/21 (28.6)	0.01^2^
Little white sputum	16/63 (25.4)	0/21 (0.0)	0.01^2^
White sputum	4/63 (6.3)	5/21 (23.8)	0.03^2^
Little yellow sputum	3/63 (4.8)	0/21 (0.0)	0.31^2^
Yellow—white sputum	2/63 (3.2)	4/21 (19.0)	0.01^2^
Yellow pus sputum	0/63 (0.0)	4/21 (19.0)	< 0.01^2^
Yellow sputum	0/63 (0.0)	1/21 (4.8)	0.08^2^
Phlegm	0/63 (0.0)	1/21 (4.8)	0.08^2^
Chest tightness and shortness of breath: Yes	8/63 (12.7)	10/21 (47.6)	< 0.01^2^
Hemoptysis: Yes	0/63 (0.0)	4/21 (19.0)	< 0.01^2^
Chills: Yes	29/63 (46.0)	7/21 (33.3)	0.31^2^
Influenza-like symptoms: Yes	31/63 (49.2)	4/21 (19.0)	0.02^2^
Gastrointestinal symptoms: Yes	10/63 (15.9)	3/21 (14.3)	0.86^2^
Neuropsychiatric symptoms: Yes	5/63 (7.9)	3/21 (14.3)	0.39^2^

N is the number of non-missing value. ^1^Kruskal-Wallis. ^2^Pearson. ^3^Wilcoxon.

### Laboratory findings

We conducted a statistical analysis of the initial laboratory test outcomes following admission for patients diagnosed with all two forms of atypical pneumonia. The complete set of patients with *C. psittaci* pneumonia (*N* = 63) and *Legionella* pneumonia (*N* = 21) were compared in Table 3.

**TABLE 3:** Laboratory tests results of 84 patients.

	*C. psittaci* pneumonia (*N* = 63)	Legionella (*N* = 21)	*p*-value
PCO2: Median (IQR)	32.0 (29.3—34.8)	35.5 (29.8—43.2)	0.20^3^
PO2: Median (IQR)	71.0 (61.2—77.2)	62.5 (55.6—71.1)	0.20^3^
Leukocytes (× 10^9^ per L; normal range 3^⋅^5–9^⋅^5): Mean ± SD	6.55 ± 2.74	11.36 ± 4.93	< 0.01^3^
Neutrophils ratio (normal range 0.4–0.75): Median ± SD	0.724 ± 1.681	0.789 ± 0.199	< 0.01^3^
Lymphocytes ratio (normal range 0.2–0.5): Median ± SD	0.167 ± 0.095	0.134 ± 0.138	< 0.01^3^
NLR: Median ± SD	6.85 ± 5.99	10.07 ± 6.00	< 0.01^3^
Rapid C-reactive protein (mg/L): Median ± SD	118.77 ± 71.17	159.36 ± 100.66	0.15^3^
Erythrocyte sedimentation (mm/h): Median ± SD	63 ± 27	46 ± 33	0.07^3^
Procalcitonin (ng/ml): Median ± SD	0.62 ± 1.53	1.06 ± 1.97	0.42^3^
Serum sodium (mmol/L): Median ± SD	135.23 ± 5.08	133.30 ± 4.94	0.08^3^
Serum potassium (mmol/L): Median ± SD	3.63 ± 0.44	3.86 ± 0.53	0.13^3^
Serum phosphorus (mmol/L): Median ± SD	0.82 ± 0.24	0.80 ± 0.37	0.25^3^
Albumin (g/L): Median ± SD	33.7 ± 4.3	32.9 ± 7.0	0.56^3^
Blood urea nitrogen (mmol/L): Median ± SD	4.57 ± 1.63	6.62 ± 6.46	0.10^3^
Creatinine (μmol/L): Median ± SD	68.7 ± 20.3	107.8 ± 154.9	0.20^3^
Alanine aminotransferase (U/L): Median ± SD	58.7 ± 54.0	64.1 ± 82.7	0.41^3^
Aspartate aminotransferase (U/L): Median ± SD	64.1 ± 70.9	55.2 ± 67.1	0.41^3^
Lactate dehydrogenase (U/L): Median ± SD	328.5 ± 172.6	295.1 ± 113.3	0.47^3^
Creatine kinase (U/L): Median ± SD	547.7 ± 1940.2	167.2 ± 233.9	0.33^3^
Blood glucose (mmol/L): Median ± SD	6.19 ± 2.28	8.95 ± 4.26	0.01^3^
Serum Ferrites (ng/ml): Median ± SD	1033.0 ± 947.3	1039.2 ± 1313.2	0.62^3^
D-dimer (μg/mL): Median ± SD	1.20 ± 2.57	1.47 ± 2.38	0.72^3^
Fibrinogen levels (g/L): Median ± SD	6.20 ± 1.63	6.60 ± 1.67	0.25^3^
BALF culture			< 0.01^2^
Negatives	59/59 (100)	13/15 (86.7)	< 0.01^2^
*A. baumannii*	0/59 (0.0)	0/15 (0.0)	
*Aspergillus polymorpha*	0/59 (0.0)	0/15 (0.0)	
*Candida glabrata*	0/59 (0.0)	1/15 (6.7)	
*Pseudomonas aeruginosa*; *Klebsiella pneumoniae*	0/59 (0.0)	1/15 (6.7)	
Blood culture: Yes (all negatives)	29/63 (46.0)	14/21 (66.7)	0.10^2^
Sputum culture			0.11^2^
No	3/63 (4.8)	2/21 (9.5)	
Negatives	0/63 (0.0)	1/21 (4.8)	
Normal flora	49/63 (77.8)	12/21 (57.1)	
*Candida albicans*	4/63 (6.3)	3/21 (14.3)	
*Klebsiella pneumoniae*	2/63 (3.2)	0/21 (0.0)	
*Filamentous fungus*	1/63 (1.6)	0/21 (0.0)	
*A. baumannii*; *Candida albicans*	1/63 (1.6)	0/21 (0.0)	
*Klebsiella pacifica*	1/63 (1.6)	0/21 (0.0)	
*Pseudomonas aeruginosa*; *Enterobacter holmesii*	1/63 (1.6)	0/21 (0.0)	
*Stenotrophomonas maltophilia*	0/63 (0.0)	1/21 (4.8)	
*Pseudomonas aeruginosa*; *Klebsiella pneumoniae*	0/63 (0.0)	1/21 (4.8)	
Klebsiella pneumoniae; *Candida krusei*	0/63 (0.0)	1/21 (4.8)	
Serum antibody testing			< 0.01^2^
No	25/63 (39.7)	5/21 (23.8)	
Negatives	36/63 (57.1)	9/21 (42.9)	
Mycoplasma pneumoniae IgM	1/63 (1.6)	0/21 (0.0)	
Legionella pneumophila serotype 1 IgM positive	0/63 (0.0)	3/21 (14.3)	
Legionella IgM antibody positive	0/63 (0.0)	1/21 (4.8)	
Legionella pneumophila IgM positive	0/63 (0.0)	2/21 (9.5)	
Electronic Bronchoscopy: Yes	59/63 (93.7)	15/21 (71.4)	< 0.01^2^
mNGS detection: Yes	56/63 (88.9)	16/21 (76.2)	0.15^2^
mNGS detection group			0.08^2^
BALF	55/63 (87.3)	15/21 (71.4)	0.09^2^
BALF; serum	1/63 (1.6)	0/21 (0.0)	0.56^2^
Sputum; serum	0/63 (0.0)	1/21 (4.8)	0.08^2^

N is the number of non-missing value. ^1^Kruskal-Wallis. ^2^Pearson. ^3^Wilcoxon. PCO_2_, partial pressure of carbon dioxide; PO_2_, partial pressure of oxygen; NLR, neutrophil-to-lymphocyte ratio.

Significant differences were observed in white blood cell count (WBC: 6.6 ± 2.7 vs. 11.4 ± 4.9, *p* < 0.001), neutrophil-to-lymphocyte ratio (NLR: 6.1 ± 5.9 vs. 9.8 ± 6.1, *p* = 0.022), and blood glucose levels (6.19 ± 2.28 vs. 8.95 ± 4.26, *p* = 0.009). Additionally, electronic bronchoscopy was more frequently performed in *C. psittaci* pneumonia patients (93.7% *vs.* 71.4%, *p* = *0.014*). No significant differences were found in other laboratory parameters, including PCO2, PO2, rapid c-reactive protein, erythrocyte sedimentation (ESR), procalcitonin (PCT), electrolytes, liver enzymes, LDH, CK, D-dimer, fibrinogen levels, or lactic acid. mNGS detection rates were comparable between groups, with BALF being the primary sample type for both cohorts. These findings highlight key hematological and diagnostic distinctions between the 2 pneumonia types.

Table 3 displayed the results of BALF cultures, blood cultures, sputum cultures, serum pathogen-specific antibodies, and mNGS in both patients. We had seen that the probability of BALF cultures and sputum cultures yielding meaningful pathogens was low and blood cultures were all negative.

### Chest CT findings

All patients underwent chest CT scans. The CT signs and distribution of patients with both categories of atypical pneumonia are presented in Table 4.

**TABLE 4:** Chest CT signs of 84 patients.

	*C. psittaci* pneumonia *N* = 63	Legionella *N* = 21	*p*-value
**Consolidation**			0.51^2^
No	1 (1.6%)	1 (4.8%)	0.41^2^
Lobar consolidation	31 (49.2%)	12 (57.1%)	0.53^2^
Pulmonary segment consolidation	25 (39.7%)	5 (23.8%)	0.19^2^
Massive consolidation	6 (9.5%)	3 (14.3%)	0.54
**Total score of consolidation:** Median (IQR)	2.0 (1.0—4.8)	2.0 (1.0—3.7)	0.69^3^
**Distribution of consolidation**			
Upper right lobe: Yes	19/63 (30.2%)	9/21 (42.9%)	0.32^3^
Right middle lobe: Yes	8/63 (12.7%)	1/21 (4.8%)	0.30^3^
Right Lower Lobe: Yes	32/63 (50.8%)	8/21 (38.1%)	0.49^3^
Upper Left Lobe: Yes	16/63 (25.4%)	6/21 (28.6%)	0.82^3^
Lower Left Lobe: Yes	28/63 (44.4%)	13/21 (61.9%)	0.31^3^
**Ground-glass opacity:** Yes	54/63 (85.7%)	19/21 (90.5%)	0.58^2^
**Total score of ground-glass opacity:** Median (IQR)	1.0 (1.0—2.0)	2.0 (1.0—3.0)	0.09^3^
**Distribution of ground-glass opacity**			
Upper right lobe: Yes	17/63 (27.0%)	8/21 (38.1%)	0.31^3^
Right middle lobe: Yes	9/63 (14.3%)	4/21 (19.0%)	0.60^3^
Right Lower Lobe: Yes	23/63 (36.5%)	8/21 (38.1%)	0.99^3^
Upper Left Lobe: Yes	15/63 (23.8%)	9/21 (42.9%)	0.05^3^
Lower Left Lobe: Yes	27/63 (42.9%)	11/21 (52.4%)	0.38^3^
**Cloudy flocculant shadows:** Yes	3 (4.8%)	0 (0.0%)	0.31^2^
**Bronchial insufflation sign:** Yes	38 (60.3%)	7 (33.3%)	0.03^2^
**Pleural effusion:** Yes	15 (23.8%)	4 (19.0%)	0.56^2^
**Pericardial effusion:** Yes	1 (1.6%)	0 (0.0%)	0.56^2^
**Empty shell:** Yes	4 (6.3%)	0 (0.0%)	0.24^2^
**Pulmonary hypotension:** Yes	1 (1.6%)	0 (0.0%)	0.56^2^
**Giddy sign:** Yes	9 (14.3%)	0 (0.0%)	0.07^2^
**Reflexology:** Yes	3 (4.8%)	2 (9.5%)	0.42^2^
**Bronchial distribution predominates:** Yes	0 (0.0%)	1 (4.8%)	0.08^2^
**Peripheral distribution predominates:** Yes	18 (28.6%)	2 (9.5%)	0.08^2^

N is the number of non-missing value. ^1^Kruskal-Wallis. ^2^Pearson. ^3^Wilcoxon.

**FIGURE 1:**
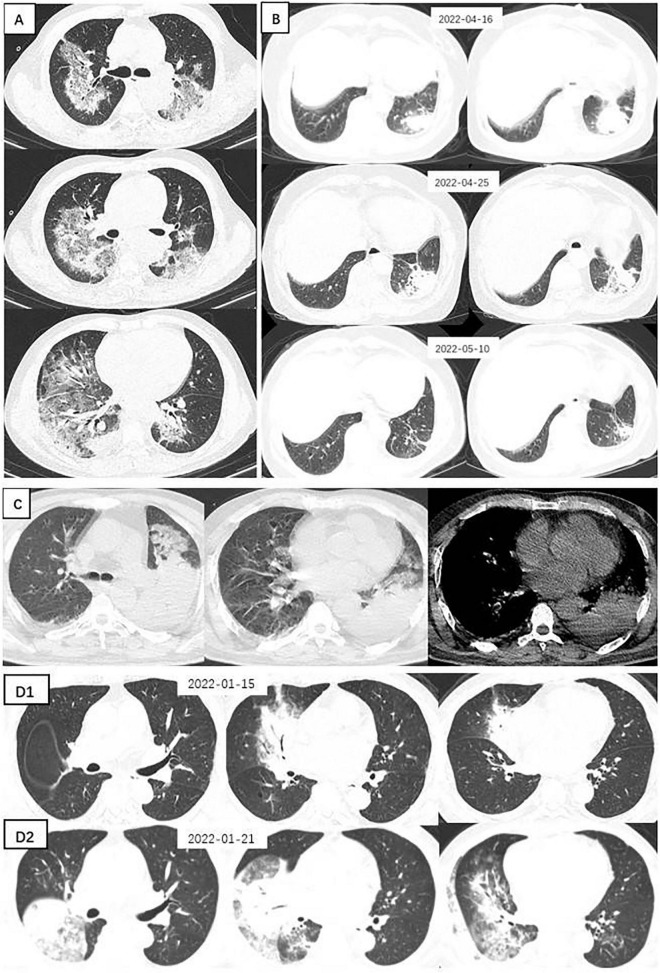
Chest CTs of 4 patients with *Chlamydia psittaci pneumonia*. **(A)** Large sheets of ground-glass shadows are seen in both lungs, within which fine latticework and small solid shadows are seen. **(B)** A spherical lesion is seen in the left lower lung, surrounded by fine grids and ground glass shadows. After 9 days of moxifloxacin treatment, the lesion was absorbed, and a follow-up CT at a further interval of 15 days showed that the lesion was largely absorbed and dissipated, leaving scattered fibrous nodular foci. **(C)** Large solid shadow in the left lower lung surrounded by ground-glass opacities and a small amount of pleural effusion. **(D)** A solid lesion in the middle lobe of the right lung, within which the bronchial inflation sign is seen, surrounded by ground glass shadows. Repeat CT 6 days later suggested progression of the disease, with a significant increase in the extent of solid lesions in the middle lobe of the right lung and real changes in the other lobes, with multiple flocculent and ground-glass opacities in several lobes of the lung and pleural effusion.

**FIGURE 2:**
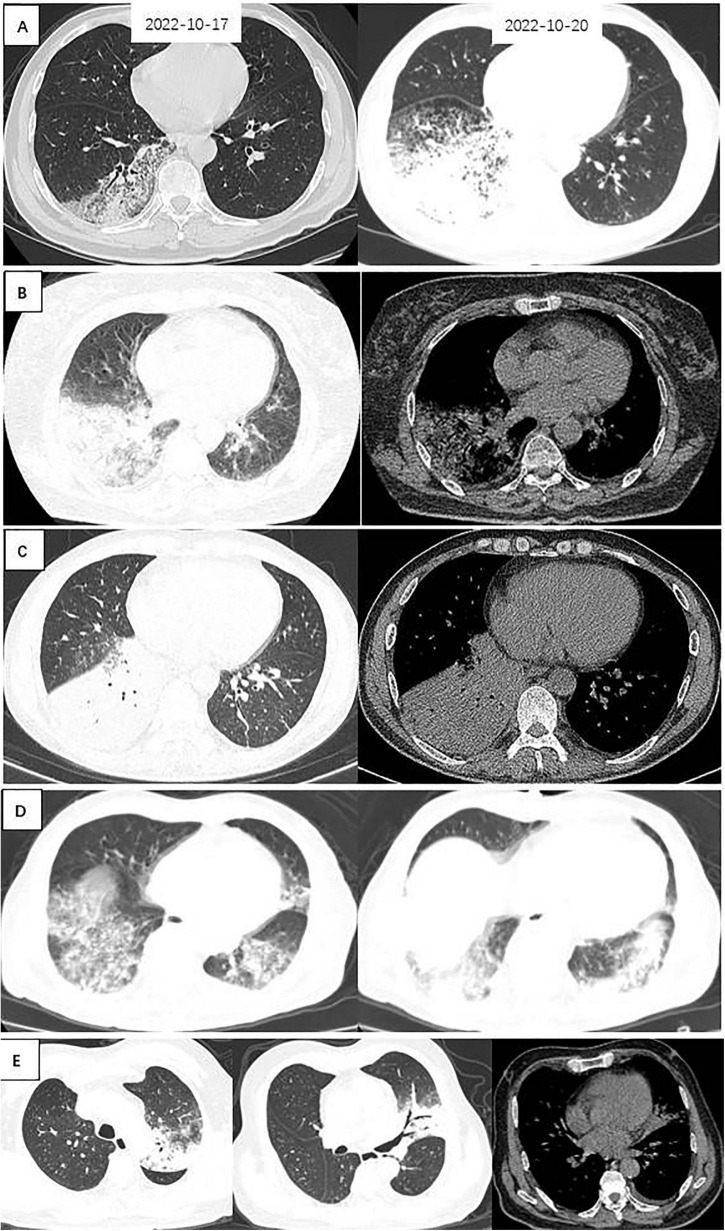
Chest CTs of 5 patients with *Legionella* pneumonia. **(A)** Patchy ground-glass density, fine latticework shadows with bronchial penetration signs are seen in the right lower lung; a repeat CT 3 days later suggested that the extent of the lesion had increased significantly, and large patches of ground-glass density and fine-mesh shadows were seen throughout the right lower lung, with lamellar solid shadows seen within them. **(B)** Large solid shadows with bronchial air phases and ground glass density shadows were seen in the right lower lung, with a small amount of pleural effusion. **(C)** A large solid shadow with bronchial air phase is seen in the right lower lung. **(D)** Patchy ground-glass densities, fine lattice shadows, and fibrosis are seen in both lower lungs. **(E)** Left upper lungs exhibit solid shadows, signs of bronchial inflation, ground glass density, and gravity drop.

Chest CT manifestations of *C. psittaci* pneumonia and *Legionella* pneumonia were the most difficult to differentiate. Their most common chest CT signs were consolidation, bronchial insufflation sign, and ground-glass opacity, which were combined with pleural effusion in some patients. Statistical analysis of the chest CT signs of the two alone revealed that *Legionella* pneumonia had a higher probability of having a ground-glass opacity in the upper left lobe than *C. psittaci* pneumonia, and the difference was statistically significant (*p* = 0.05). However, bronchial insufflation signs were more frequent in *C. psittaci* pneumonia, and again, the difference was statistically significant (*p* = 0.03). Other signs were not statistically different when comparing these 2 types of pneumonia. Figure 1 illustrates *C. psittaci* pneumonia, whereas Figure 2 illustrates *Legionella* pneumonia.

### Treatment and outcome

All patients received antibiotic treatment (Table 1). Patients often received combination treatments before diagnosis. In patients with *C. psittaci* pneumonia, *and Legionella* pneumonia, carbapenem antibiotics were used no less frequently: 20/63 (31.7%), 14/21 (66.7%), respectively, and *p* = 0.01. Before diagnosis, a significant proportion of patients (69.8%, 80.9%; respectively) received two or more antibiotics simultaneously (*p* = 0.32). Some patients received more than four antibiotics, particularly in patients with *Legionella* pneumonia (23.8%) (*p* = 0.01). However, once the pathogenic bacteria had been identified, antibiotic regimens were often adjusted to include only doxycycline, oxifloxacin, or nemonoxacin malate.

As shown in Table 1, about 66.7% of patients with *Legionella* pneumonia had comorbid ketosis, but only 33.3% had diabetes. In addition, the incidence of ketosis and previous history of diabetes were higher than those of *C.* psittaci pneumonia (*p* = 0.02, *p* = 0.05). Patients with *C. psittaci* pneumonia and *Legionella* pneumonia were more likely to have a combination of complications: respiratory failure, abnormal liver function, hypoalbuminemia, and electrolyte disturbances. And they were more likely to develop respiratory failure and even ARDS. Many patients with *Legionella* pneumonia had a higher proportion of severe cases and respiratory failure, longer hospital stays, and one patient died (*p* < 0.01, *p* = 0.01, *p* = 0.08). However, there was no significant difference in imaging uptake time among these two pneumonia types.

### Distinguishing between *C. psittaci* pneumonia and *Legionella* pneumonia

**FIGURE 3:**
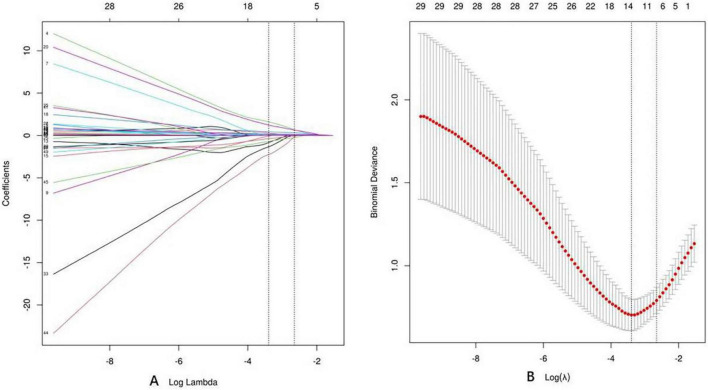
Lasso regression. **(A)** Coefficient profiles for Lasso regression. **(B)** Lasso regression cross-validation plot.

First, we used LASSO regression to identify variables that differentiate *C. psittaci* pneumonia from *Legionella* pneumonia. Lasso regression was modeled by binomial, where λ *min* = 0.044, λ *1se* = 0.077. We chose λ *1se* = 0.077, which corresponds to the choice of variables for the model: cough, chest tightness and shortness of breath, upper left lobe (distribution of ground-glass opacity), bronchial distribution predominates, leukocytes (WBC), *l* CK (the natural logarithm of CK), blood glucose (GLU). Figure 3A shows the Lasso regression coefficient profiles, and Figure 3B shows the Lasso regression cross-validation plot.

**FIGURE 4:**
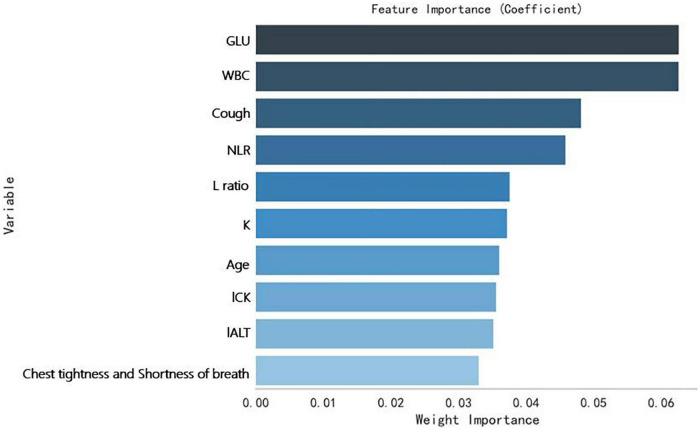
The weights of variables importance.

Secondly, a Random Forrest Classifier was used to analyze the importance of the variables. The 10 most important variables (from highest to lowest) were obtained: GLU, WBC, Cough, NLR, L ratio (lymphocytes ratio), K (Serum potassium), age, *l* CK, *l* ALT (the natural logarithm of ALT), Chest tightness and shortness of breath. Figure 4 shows them.

**FIGURE 5:**
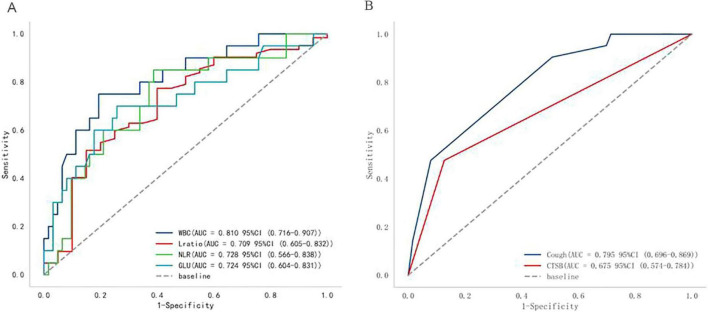
ROC plots of the variables (**A** for numerical variables, **B** for categorical variables). WBC, Leucocytes; L ratio, Lymphocytes ratio; NLR, neutrophil-to-lymphocyte ratio; GLU, Blood glucose; CTSB, Chest tightness and shortness of breath.

Finally, we analyzed all variables screened by LASSO regression and Random Forrest Classifier through ROC curves. Table 5 shows the results of the ROC curve analysis for each variable. There were six statistically significant variables among them: four continuous variables: WBC, L ratio, NLR, and GLU, as shown in Figure 5A; and two categorical variables: cough, chest tightness and shortness of breath, as shown in Figure 5B.

**TABLE 5:** ROC curve results for variables.

Characteristics	AUC	Youden’s index	Cutoff	Sensitivity	Specificity	95%CI	*P*
Age	0.547	0.197	70.000	0.350	0.847	0.378–0.710	0.550
Leukocytes	0.810	0.547	8.280	0.750	0.797	0.716–0.907	< 0.001
NLR	0.728	0.443	5.141	0.850	0.593	0.566–0.838	0.008
Lymphocytes ratio	0.709	0.363	0.102	0.763	0.600	0.605–0.832	0.003
Serum potassium	0.634	0.297	4.070	0.450	0.847	0.466–0.757	0.119
Creatine kinase	0.586	0.222	266.4	0.322	0.900	0.428–0.717	0.324
Blood glucose	0.724	0.429	6.630	0.700	0.729	0.604–0.831	0.006
Alanine aminotransferase	0.544	0.214	18.4	0.814	0.400	0.401–0.721	0.410
Cough	0.795	0.400	3.000	0.900	0.500	0.696–0.869	< 0.001
Chest tightness and shortness of breath	0.675	0.349	1.000	0.476	0.873	0.574–0.784	0.017
Upper left lobe	0.615	0.191	1.000	0.429	0.762	0.497–0.742	0.115
Bronchial distribution redominates	0.524	0.048	1.000	0.048	1.000	0.500–0.583	0.745

ROC curve, receiver operating characteristic curve; AUC, the area under the ROC curve; CI, confidence interval; NLR, neutrophil-to-lymphocyte ratio.

The factors that could identify *C. psittaci* pneumonia and *Legionella* pneumonia with good discriminatory ability were found by LASSO regression, a Random Forrest Classifier, and ROC curves. These factors were WBC, lymphocyte ratio, NLR, GLU, cough, chest tightness and shortness of breath; with respective AUC’ s of 0.810, 0.709, 0.728, 0.724, 0.795, 0.675; 95% CI’ s of 0.716–0.907, 0.605–0.832, 0. 566–0.838, 0.604–0.831, 0.696–0.869, 0.574–0.784; and all were statistically significant (*P* < 0.001, 0.003, 0.008, 0.006, < 0.001, 0.017; respectively).

When cough was combined with chest tightness and shortness of breath, its AUC was 0.844, *r* = 0.555, 95% *CI* 0.747–0.940, *P* < 0.001; when cough with chest tightness and shortness of breath, was combined with lymphocytes ratio reduction, its AUC was 0.891, *r* = 0.651, 95% *CI* 0.818–0.964, *P* < 0.001; when cough with chest tightness and shortness of breath, was combined with hyperglycemia, its AUC was 0.877, *r* = 0.68, 95% *CI* 0.784–0.970; when cough with chest tightness and shortness of breath with lymphocytes ratio reduction and hyperglycemia, its AUC was 0.896, *r* = 0.665, 95% *CI* 0.820–0.973, *P* < 0.001. All of the above suggest that *Legionella* is more likely.

## Discussion

This is an extended retrospective study of the epidemiology and clinical characteristics of atypical pathogens (*C. psittaci*, and *Legionella*). A total of 84 patients were studied at Wuxi Second People’s Hospital. It presents the current status of atypical pathogen infections in Wuxi. In this paper, we summarize the clinical characteristics of the two atypical pathogens and search for their points of differentiation. Particularly, distinguishing factors between *C. psittaci* pneumonia and *Legionella* pneumonia were explored.

In our study, *C. psittaci* had a history of mostly avian exposure, and *Legionella* pneumophila had a history of three specific water-related exposures (facial spas, swimming in public baths, drowning). The results of this epidemiological history are similar to those of previous studies and will not be discussed in detail here (3, 16).

As we all know, *C. psittaci* pneumonia and *Legionella* pneumonia are rare. Based on 57 studies published before 2012, *C. psittaci* caused 1.03% of CAP cases (3). Recent decades have observed an increase in *C. psittaci* and *Legionella* pneumonia incidence rates. According to a recent multicenter study in China, *C. psittaci* infection reached 6.8%, and *Legionella pneumophila* reached 11.3% (2). A retrospective single-center study reported 16 cases of severe psittacosis pneumonia in 2019–2020 (17). And there were 55 *C. psittaci* pneumonia cases reported in Wuxi during 2020–2022 alone (5). In Europe and North America, *Legionella* pneumonia accounts for about 2–15% of all community-acquired pneumonia (16). In addition to their increased incidence, all both pathogens can cause severe pneumonia, and in severe cases, fatal cases can occur. Therefore, we explore the clinical features of these two types of pneumonia to deepen our understanding of them for early diagnosis and treatment.

We know that the most common symptoms of *C. psittaci* pneumonia include fever, high temperature, cough, and sputum (6, 17). Similarly to *C. psittaci* patients, the clinical symptoms of *Legionella* pneumonia cases presented with fever (≥ 39°C) (100%), cough (100%), dyspnea (100%), asteria (3/4, 75%), shivering (4/4, 100%), headache (4/4, 100%), diarrhea (3/4, 75%) and abdominal distension or pain (3/4, 75%), and phlegm was uncommon (18). In our study, we found that the main clinical manifestations of patients with *C. psittaci* pneumonia were high fever, cough, chills, and Influenza-like symptoms. Patients with *Legionella* pneumonitis primarily present with high fever and severe cough with sputum production, chest tightness and shortness of breath.

The clinical features of these patients in this retrospective study were not the same, but they shared many symptoms. Almost all had fevers. In our study, we found differences in their clinical presentation. *C. psittaci* pneumonia presented with high fever; 20.6% of patients had no cough, 28.6% had no severe cough (cough score 1 – 2), and 60.3% had no sputum. However, patients with *Legionella* pneumonia not only had high fever but also severe cough (≥ 3 cough score in more than 90.5%), and cough was also one of the discriminators between *C. psittaci* pneumonia and *Legionella* pneumonia, with AUC = 0.795 and a *CI* of 0.696–0.869. There are no findings reported in previous studies.

Patients with atypical pneumonia were previously thought to have the following characteristics: typical patient history, lack of purulent sputum, typical chest X-ray findings, absence of leukocytosis, normal or moderately elevated C-reactive protein, and poor response to β-lactam therapy (18). However, in our study, *Legionella* pneumonia leukocytes and CRP were significantly elevated with mean ± *SD* of (11.36 ± 4.93) × 10^9^/L and 159.36 ± 100.66 mg/. Up to 71.4% of patients had sputum, and 42.8% had yellow sputum. CRP was significantly elevated in patients with *C. psittaci* pneumonia, up to (118.77 ± 71.17) mg/L. However, these symptoms and signs do not easily distinguish “atypical” from “typical” disease. They overlap, and even a lobar infiltrate on chest CT may be caused by an atypical pathogen. In both types of atypical pneumonia, consolidation, ground-glass opacity, and bronchial insufflation are common signs. Typical pneumonia also exhibits this characteristic. In light of this, we believe it is necessary to explore the clinical characteristics of various atypical pneumonia cases carefully.

As we know, in *C. psittaci* pneumonia, high fever is a characteristic clinical feature. There is a significant increase in the neutrophil ratio, NLR, rapid C-reactive protein, CK, and LDH in patients with normal leucocyte counts, while the ratio of lymphocytes, albumin, serum sodium, potassium, and phosphorus are decreased. Chest CT shows consolidation, bronchial inflation sign, ground glass opacities, etc. (5, 19). According to this study, previous research perspectives have been supported. Previously, *Legionella* pneumonia was believed to have non-specific clinical and imaging manifestations, and some clinical and laboratory abnormalities were associated with *Legionella* diagnosis. The abnormalities include hyponatremia, hypophosphatemia, elevated liver enzymes, altered mental state, headache, diarrhea, and acutely elevated creatine phospholipase levels (16, 20). In our study, we found that patients with *Legionella* pneumonia not only have high fever but also severe coughing. They had the highest levels of leukocytes, and NLR; which were statistically significant.

Both *Legionella* pneumonia and *C. psittaci* pneumonia exhibit a number of similarities in clinical manifestations, laboratory tests, and chest CT findings (21). In contrast, *Legionella* pneumonia has a higher level of serious illness and mortality. The severe illness rate of *Legionella* pneumonia reached 71.4% in this study, and one person died. According to an epidemiological survey in Japan, *Legionella* pneumonia is associated with a 6.4% mortality rate (22). It is challenging to distinguish *C. psittaci* pneumonia from *Legionella* pneumonia. In this article, we explore their discriminatory points using statistical methods.

In this study, we combined LASSO regression and ROC curve analysis to explore the factors that could identify the differences between *C. psittaci* pneumonia and *Legionella* pneumonia. These factors were WBC, Lymphocytes ratio, blood glucose, cough, chest tightness and shortness of breath, influenza-like symptoms, ketone bodies, and complications. Patients with *Legionella* pneumonia may exhibit a more severe cough, chest tightness and shortness of breath. They may also have higher leukocytes, NLR, blood glucose, and a lower percentage of lymphocytes. The reverse tendency may be *C. psittaci*. It differs from previous reports, which indicated that *C. psittaci* typically presents with a dry cough, pleurisy-related chest pain, and difficulty breathing (16). However, this study suggests that *Legionella* pneumonia is more likely to present with severe coughing, chest tightness and shortness of breath. Of course, we also need to combine the chest CT findings of patients. Their most common chest CT signs are consolidation, bronchial inflation signs, and ground glass opacities, each with its characteristic. For example, the probability of ground glass opacities appearing in the upper left lobe of *Legionella* pneumonia is higher than that of *Chlamydia psittaci* pneumonia. In addition, bronchial inflation signs are more common in *C. psittaci* pneumonia, which is statistically significant. Previous reports were generalized and did not further compare the differences between their clinical presentations and laboratory findings. This discovery will help clinical doctors deepen their understanding of these two types of pneumonia.

In our study, an issue that should not be overlooked was the concomitant use of two or more antibiotics in 69.8%, 80.9% of patients with *C. psittaci* pneumonia, *Legionella* pneumonia, respectively. The frequency of carbapenem antibiotics was equally high among the combinations: 31.7%, 66.7%, respectively. Some patients even received more than four antibiotics. The uncertainty of diagnosis was considered a reason for the increased use of broad-spectrum antibiotics. The indiscriminate use of various antibiotics is problematic. They are the driving factors behind the increasingly serious problem of antibiotic resistance (AMR) worldwide (23). The World Health Organization lists AMR as a major global threat. Third generation cephalosporins, macrolides, fluoroquinolones, piperacillin/tazobactam, and carbapenems are key antibiotics for humans, and their unnecessary use should be restricted to avoid resistance (24). But in our study, once the pathogenic bacteria had been identified, the antibiotics were often adjusted so that only doxycycline, moxifloxacin, or nemonoxacin malate were used. It again emphasizes the importance of gaining a thorough understanding of both types of pneumonia. The goal is to identify pathogens as soon as possible and diagnose them as soon as possible.

There are several limitations to this study: (1) Among the 84 patients included, there were only 21 cases of *Legionella* pneumonia, so *Legionella* pneumonia could not be thoroughly analyzed. (2) Pathogens in individual cases were identified through pathogen-specific antibodies and not by mNGS. (3) The current study focused on the overall situation of both types of atypical pneumonia without analyzing and comparing mild and severe cases.

## Conclusion

This study analyzed *C. psittaci* pneumonia, and *Legionella* pneumonia from multiple perspectives, exploring their clinical characteristics and unique features. A challenge lies in distinguishing *C. psittaci* pneumonia from *Legionella* pneumonia. There were statistically significant differences between them in this study, with *Legionella* pneumonia having a clinical manifestation of severe coughing and more patients experiencing chest tightness and shortness of breath. During laboratory tests, *Legionella* pneumonia had a higher level of leukocytes, NLR, and blood glucose while having a lower proportion of lymphocytes. These findings are helpful for early diagnosis of diseases, beneficial for antibiotic selection, and avoiding unreasonable and unnecessary use of antibiotics.

The study provides actionable guidance to optimize therapy in the following key clinical scenarios:

Scene 1: Guidance for early antibiotic adjustment prior to mNGS results: When mNGS results are pending (usually 24–72 h), and in patients with CAP with multisystemic involvement and multiple imaging signs such as chest CT suggesting multilobar, multisegmental consolidation with bronchial congestion, ground-glass opacities, pleural effusions, etc., atypical pathogens should be considered, and antibiotic coverage of atypical pathogens is warranted. This may include choosing macrolides or quinolones. In case of severe CAP, combine with ß-lactams empirically. Avoid triple or more antibiotics, and avoid carbapenems.

Scene 2: The following three key clinical scenarios are present in the above patients: 1) Severe cough + chest tightness and shortness of breath + lymphocytes ratio is more reduction/hyperglycemia, tend to *Legionella*, prefer quinolone antibiotics such as moxifloxacin; 2) A mild cough or no cough + lymphocyte ratio is not decreased obviously/without hyperglycemia, tend to *C. psittaci*, and prefer macrolides such as doxycycline; 3) Keep experience covering atypical pathogens until mNGS results are available.

Scene 3: If it is impossible to perform tracheoscopy or mNGS test due to conditions, refer to scene 1 and 2 for empirical coverage of atypical pathogens, preferring quinolones or macrolides according to scene 2.

## Data Availability

The original contributions presented in the study are included in the article/supplementary material, further inquiries can be directed to the corresponding author/s.
